# Case Report of a Patient With Malignant Peripheral Nerve Sheath Tumor Treated With Wide Resection

**DOI:** 10.7759/cureus.49055

**Published:** 2023-11-19

**Authors:** Arın Celayir, Mete Özer, Şeyhmus Kavak, Mahmut Kursat Ozsahin, Huseyin Botanlioglu

**Affiliations:** 1 Orthopaedics and Traumatology, Istanbul University-Cerrahpasa, Cerrahpasa Medical Faculty, Istanbul, TUR

**Keywords:** marble-like pattern, radiotherapy, wide resection, malignant schwannoma, malignant peripheral nerve sheath tumor (mpnst)

## Abstract

Malignant peripheral nerve sheath tumors are soft tissue sarcomas originating from peripheral nerves. They are more frequently diagnosed in individuals with neurofibromatosis and tend to affect young men more often than women. The most common sites for these tumors within the peripheral nerve sheath are in the pelvis and the distal femur.

Although chemotherapy and radiotherapy are not frequently used, it should be noted that in some cases, postoperative radiotherapy and chemotherapy may be beneficial. The primary treatment approach typically involves the complete surgical removal of the tumor. Here, we discuss the case of our patient whom we successfully treated with extensive resection and followed up with postoperative radiotherapy at our clinic.

## Introduction

Malignant peripheral nerve sheath tumors, originating from peripheral nerves, account for approximately 3% to 5% of all soft tissue sarcomas. They are more prevalent in individuals with neurofibromatosis and tend to occur more frequently in young men than in women [1].

These tumors are most commonly found in the gluteal region, pelvis, and axilla. In histopathological examinations, they typically appear as large, spindle-shaped, or eccentric masses within the nerve. The cells exhibit spindle shapes with nuclei resembling commas and have indistinct cytoplasm. Microscopic findings may reveal a marbled pattern [2]. S-100 protein is commonly present in most cases, and glial fibrillary acidic protein can be detected in about 30% of cases. Metastasis most commonly spreads to the lungs, liver, and lymph nodes. The primary treatment approach typically involves extensive resection. In patients with neurofibromatosis, the overall five-year survival rate is approximately 30%, whereas in sporadic cases, it is around 75%. Prognosis is less favorable in neurofibromatosis patients due to the tumor’s involvement in the trunk and extremities. Radiotherapy and chemotherapy have limited effectiveness in treatment [3].

In this article, we discuss a patient who was admitted to our hospital with a malignant peripheral nerve sheath tumor in the groin region of the right leg. We performed wide resection surgery and had a good postoperative follow-up period of two years.

## Case presentation

A 54-year-old male patient was admitted to our hospital with right thigh pain. The patient described experiencing stiffness and night pains in the right groin region for the past four months. During the physical examination, both lower extremities exhibited a full range of motion without pain. A visible mass was observed on the medial aspect of the right thigh. The patient reported pain only when palpated around the medial edge of the groin and medial femur. The patient had no other systemic diseases and was not taking any medications. He had not undergone chemotherapy or received any radiotherapy treatments.

**Figure 1:**
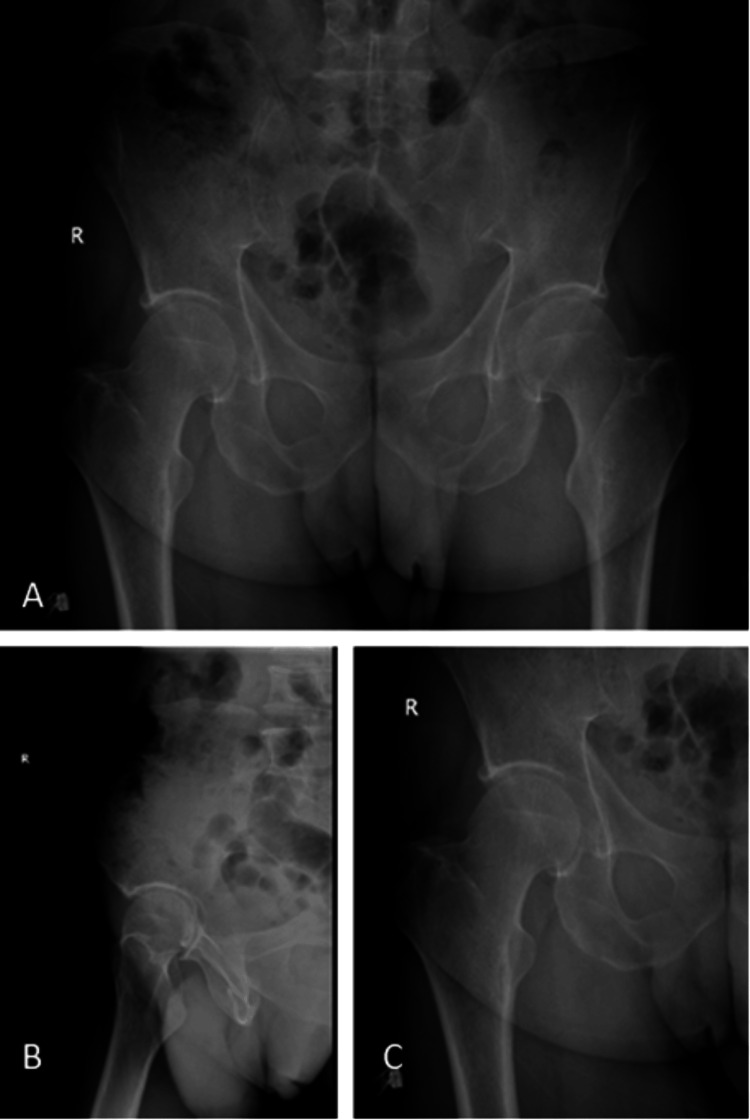
Preoperative radiological images of the patient at the admission. The pelvis anteroposterior (A), the right hip lateral (B), and the right hip anteroposterior (C) views can be seen.

**Figure 2:**
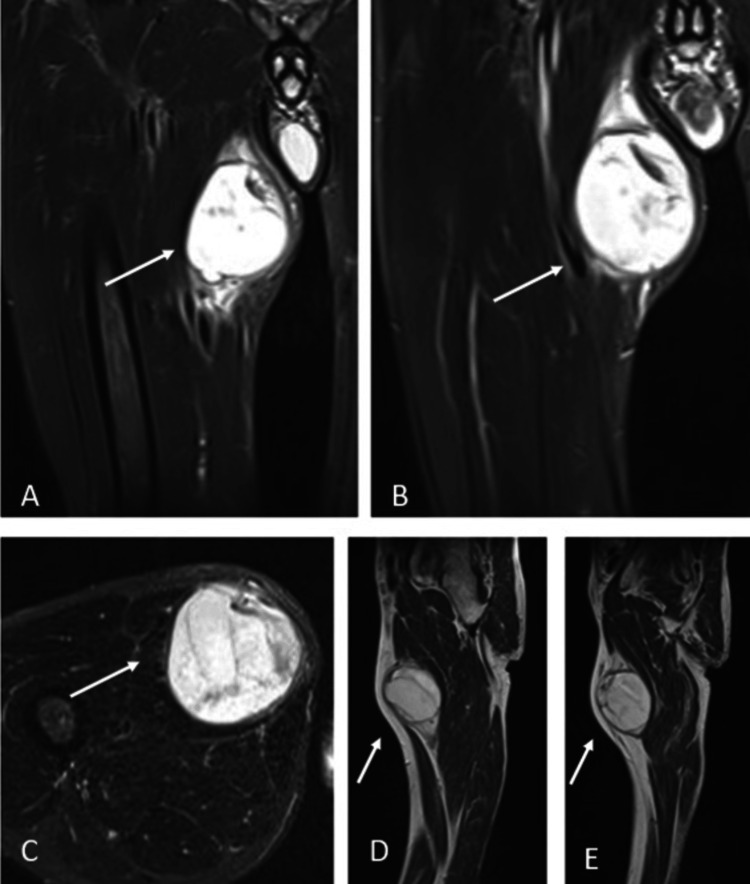
Preoperative magnetic resonance image of the patient can be seen in different sections. The lesion was 60 x 63 x 72 mm in scale. The white arrow shows the lesion in the relevant magnetic resonance images.

He came to our hospital after undergoing the initial evaluation through magnetic resonance imaging and a whole-body positron emission tomography/computerized tomography scan at another medical facility. The magnetic resonance imaging showed a lesion in the right thigh measuring 60 x 63 x 72 mm, located within the adductor longus muscle. Before any medical intervention was initiated, the patient’s consent was obtained. Direct radiological imaging was performed, and the findings from the previous magnetic resonance imaging and positron emission tomography/computerized tomography scan were discussed in our multidisciplinary tumor council (Figures 1, 2). The imaging did not reveal any metastases, and the lesion’s standardized uptake value was 5.7.

**Figure 3:**
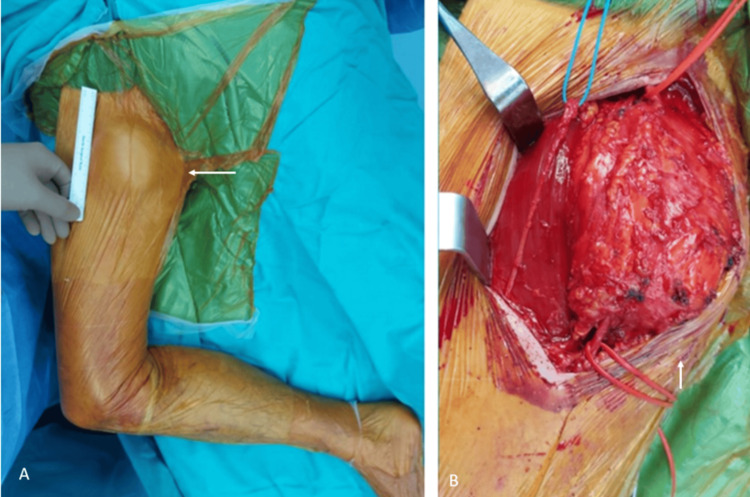
Peroperative photos of the patient. The lesion can be seen in the medial aspect of the right thigh before (A) and after the surgery (B). The white arrow shows the lesion during surgery.

**Figure 4:**
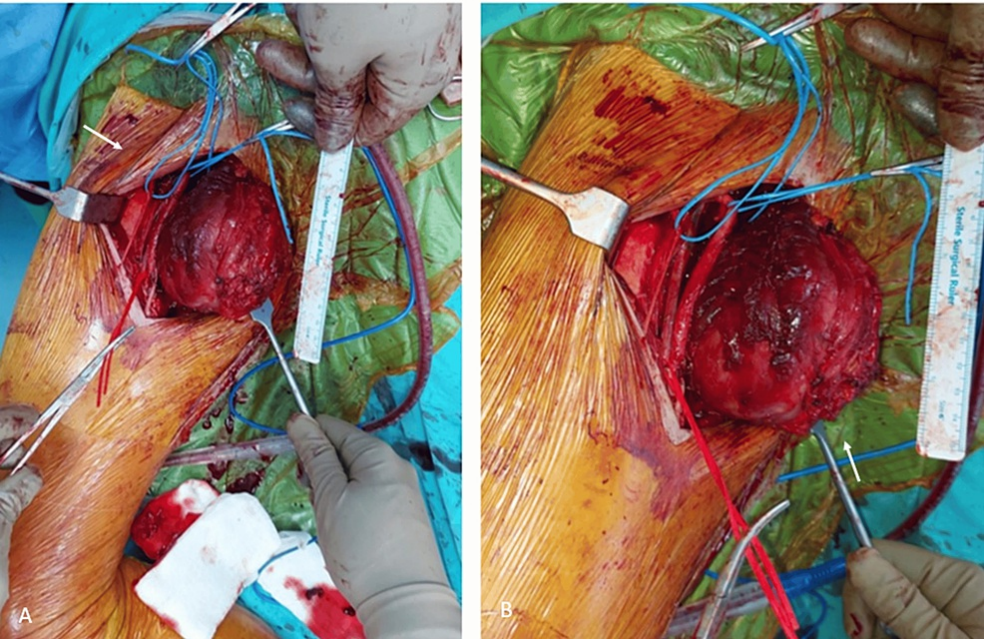
The lesion was separated from the femoral arterial branches (A and B), and there were no direct involvement of the neurovascular structures. The white arrow shows the lesion during surgery.

**Figure 5:**
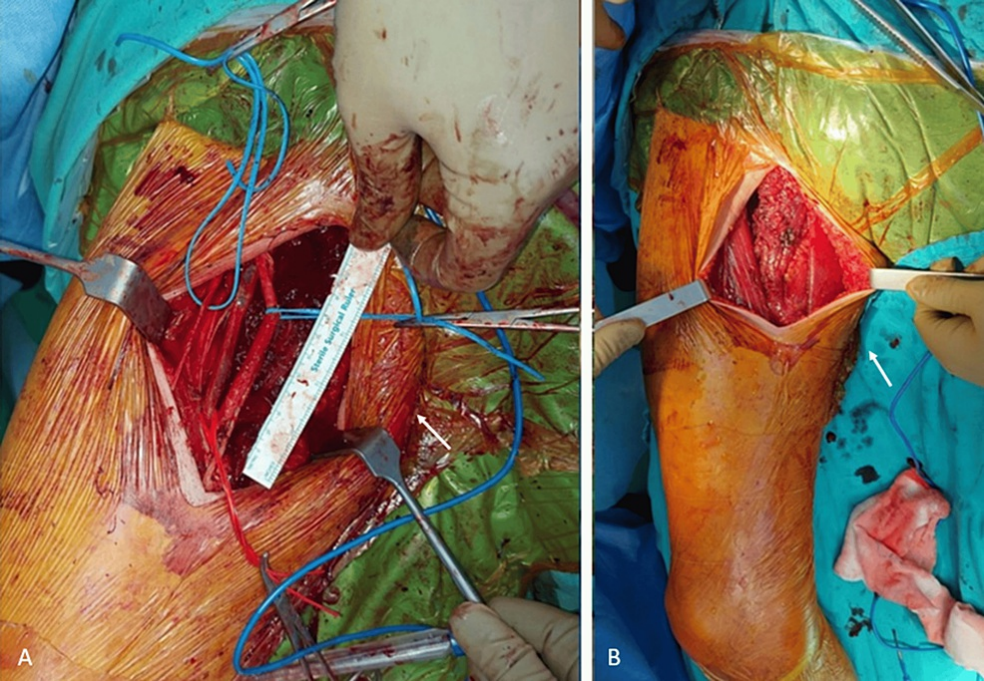
The view of the right thigh after the removal of the lesion (A). The appearance before closing the incision line with the remaining muscles (B). The white arrow is showing the field after surgical excision.

**Figure 6:**
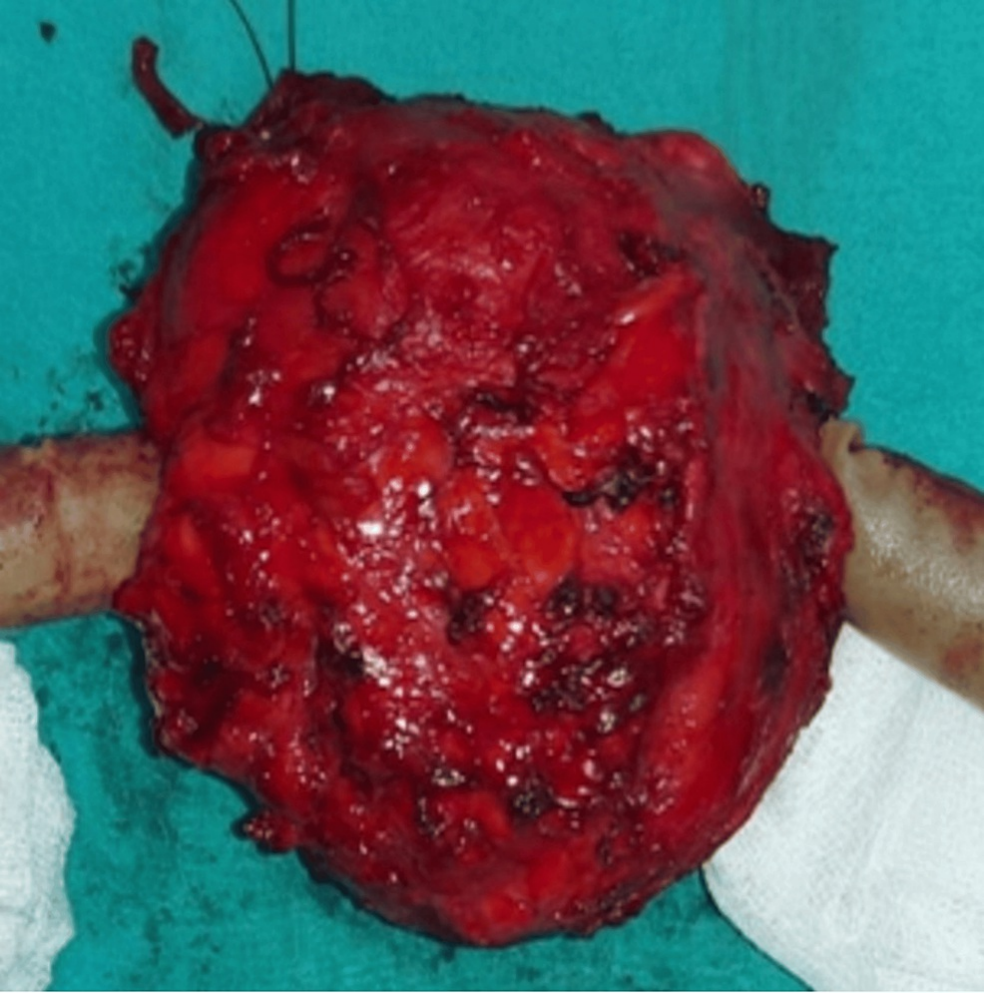
The lesion can be observed after the removal with wide resection.

After initial evaluations, we performed a wide resection surgery. During the surgery, the lesion was located inside the adductor longus muscle (Figure 3). In the center of the tumor tissue, we identified and isolated the superficial femoral artery and vein. A nerve believed to be a branch of the femoral nerve, located in the medial aspect of the lesion, was preserved and separated from the tumor tissue. The nerve tissue that had branched into the tumor lesion was excised and left within the tumor tissue. We ligated and excised the artery that entered from the proximal end of the tumor tissue and exited from the distal end. Approximately 2 cm of the neighboring area of the adductor longus muscle, inferior to the tumor tissue, was included in the resection. The lesion, along with the surrounding fascia and muscular tissue, was excised as a single block. Peroperative frozen samples were sent, and the surgical margins were negative (Figures 4-6). The pathology result was “The tumor cells are SMA (-), TLE1 (patchy +), EMA (-), MUC-4 (-), Desmin (+ in rare cells), S100 (-), CD34 (+), SOX10 (-), PanTRK (-), STAT-6 (-). Expression loss was observed with H3K27Me3. The Ki67 proliferation index is 20%.”

After the wide resection surgery, the wound site remained clear, and there was no leakage. The patient was discharged from the hospital after a week and scheduled for weekly postoperative check-ups. Approximately one and a half months later, the patient underwent 20 sessions of radiotherapy at the Radiological Oncology Department. Following the radiotherapy, the patient returned for another checkup, and the wound site remained clear. The patient had no symptoms after the combined therapy. After a two-year follow-up, there were no active symptoms, and no recurrence was observed in the control magnetic resonance images and whole-body positron emission tomography/computerized tomography scan.

## Discussion

Malignant peripheral nerve sheath tumors are neoplasms that can manifest in various parts of the body and are known for their aggressiveness, with metastasis not uncommon. The lungs are the most prevalent site for metastasis [4]. These tumors are more frequently encountered in young males and are typically observed in the pelvis or the distal femur regions of the lower extremities. In our specific case, the tumor’s origin within the adductor longus muscle and its extension into the proximal femur marked an unusual location compared to what is typically expected. Additionally, the patient presented with the tumor at a later age than the typical age of onset [5].

The five-year survival rate for individuals diagnosed with malignant peripheral nerve sheath tumors is typically in the range of 23% to 69%. The life expectancy of someone with a malignant peripheral nerve sheath tumor is influenced by factors such as the tumor’s size and its location within the body. Generally, individuals with smaller tumors tend to have a longer life expectancy compared to those whose cancer has metastasized to other parts of the body [6]. In our case, the patient did not have any metastasis, but the size of the tumor was approximately 60 x 62 x 73 cm in the magnetic resonance images. Due to the absence of metastasis and the large size of the lesion, we chose to perform a surgical wide resection followed by radiotherapy.

Chemotherapy is typically not employed in the treatment of this tumor, and radiation therapy is selectively used in specific cases. Broad resection is generally the preferred treatment approach. Following the tumor board’s decision, we conducted a wide resection and administered postoperative radiotherapy to our patient. Once the wound site had healed, the patient underwent 20 sessions of radiation therapy. As there were no metastases, chemotherapy was not prescribed. However, chemotherapy might be considered in situations where the tumor size is substantial or metastases are present [7].

## Conclusions

Malignant peripheral nerve sheath tumors are rare and malignant. They can exhibit aggressiveness, and the primary treatment approach remains the broad resection of the lesion. In general, chemotherapy does not play a significant role in the treatment, and radiotherapy may be considered in specific cases. Additionally, due to their rarity and malignant nature, early detection and timely intervention are crucial in managing these tumors effectively. Close monitoring and follow-up care are essential to assess any potential recurrence or metastasis, ensuring comprehensive and holistic patient care.
